# Robustness of radiomic features in ^123^I-ioflupane-dopamine transporter single-photon emission computer tomography scan

**DOI:** 10.1371/journal.pone.0301978

**Published:** 2024-04-11

**Authors:** Viktor Laskov, David Rothbauer, Hana Malikova

**Affiliations:** Department of Radiology and Nuclear Medicine, Third Faculty of Medicine, Charles University and University Hospital Kralovske Vinohrady, Prague, Czech Republic; Hokkaido University: Hokkaido Daigaku, JAPAN

## Abstract

Radiomic features are usually used to predict target variables such as the absence or presence of a disease, treatment response, or time to symptom progression. One of the potential clinical applications is in patients with Parkinson’s disease. Robust radiomic features for this specific imaging method have not yet been identified, which is necessary for proper feature selection. Thus, we are assessing the robustness of radiomic features in dopamine transporter imaging (DaT). For this study, we made an anthropomorphic head phantom with tissue heterogeneity using a personal 3D printer (polylactide 82% infill); the bone was subsequently reproduced with plaster. A surgical cotton ball with radiotracer (^123^I-ioflupane) was inserted. Scans were performed on the two-detector hybrid camera with acquisition parameters corresponding to international guidelines for DaT single photon emission tomography (SPECT). Reconstruction of SPECT was performed on a clinical workstation with iterative algorithms. Open-source LifeX software was used to extract 134 radiomic features. Statistical analysis was made in RStudio using the intraclass correlation coefficient (ICC) and coefficient of variation (COV). Overall, radiomic features in different reconstruction parameters showed a moderate reproducibility rate (ICC = 0.636, p <0.01). Assessment of ICC and COV within CT attenuation correction (CTAC) and non-attenuation correction (NAC) groups and within particular feature classes showed an excellent reproducibility rate (ICC > 0.9, p < 0.01), except for an intensity-based NAC group, where radiomic features showed a good repeatability rate (ICC = 0.893, p <0.01). By our results, CTAC becomes the main threat to feature stability. However, many radiomic features were sensitive to the selected reconstruction algorithm irrespectively to the attenuation correction. Radiomic features extracted from DaT-SPECT showed moderate to excellent reproducibility rates. These results make them suitable for clinical practice and human studies, but awareness of feature selection should be held, as some radiomic features are more robust than others.

## Introduction

Radiomics is a rapidly evolving field of research with significant potential in clinical medical practice regarding disease classification and patient outcome prediction. Radiomics image analysis includes the extraction of quantitative metrics, so-called radiomic features. Subsets of features indicate a “fingerprint”, a digital image phenotype of the target disease [1]. After the radiomic features are selected, they are usually used to predict target variables such as the absence or presence of a disease, treatment response, or time to symptom progression [1]. One of the potential clinical applications is in patients with Parkinson’s disease (PD).

PD is a degenerative movement disorder that is characterized by dopaminergic terminal loss in the basal ganglia and loss of dopamine-producing neurons in the substantia nigra [2, 3]. Clinical trials with neuroimaging have demonstrated the challenges in the detection of early-stage PD and establishing of the disease’s progression biomarkers [4–6]. Single photon emission tomography (SPECT) imaging of the dopaminergic system with ^123^I-ioflupane-dopamine transporter (DaT) is now widely used [7]. This imaging technique plays a critical diagnostic role, as proven by its ability to differentiate neurodegenerative Parkinsonian syndromes (PS) characterized by nigrostriatal cell loss such as PD, multiple system atrophy, and progressive supranuclear palsy from essential tremor, a movement disorder without nigrostriatal cell loss [8]. Additionally, its diagnostic application has been extended to differentiate between patients with suspected dementia with Lewy bodies and those with other subtypes of dementia [9].

PD metrics present significant issues and uncertainty. Because of the delicate and often vague nature of early PS, disease duration is a particularly challenging statistic. The ability of patients to notice the earliest signs varies considerably [3]. They are impacted by several factors, such as personality, education level, professional background, and the type of initial symptom (e.g., tremor vs. bradykinesia) [10].

Visual interpretation of SPECT DaT scans is common and uses scoring systems or calculation of the specific binding ratio, an index to measure density for DaT [7]. However, it is difficult to distinguish between age-related reductions and pathological decreases in DaT availability [11]. Unfortunately, the interpretations and measures that are not highly reliable might occasionally be found in clinical practice [12]. Thus, textural information could improve the ability to capture the disease state as manifested in the form of uneven loss of tracer uptake within the basal ganglia [13]. Despite the availability of many radiomic studies, there are few settings where radiomics is used to direct clinical decision-making. Partly because the processes for extracting radiomic measurement data are not standardized, and there is insufficient evidence of their adequate clinical validity and utility. The workflow of acquiring and processing the source images and extracting radiomic measurements should be established and harmonized [14]. One of the challenging problems for translating radiomics-based interpretation into clinical decision support systems could be its robustness. That is why it is necessary to evaluate the robustness of radiomics-based models and their potential generality [15]. The robustness of radiomic features is threatened by its sensitivity to acquisition parameters or image reconstruction algorithm variations. Few studies on the robustness of radiomic features have been made; however, they aimed at positron emission tomography (PET) imaging methods [16–22].

This study aims to determine how radiomic features extracted from DaT-SPECT images are affected by the number of iterations used in SPECT iterative reconstructions and compare this with data reconstructed using CT attenuation correction (AC) or filtered back projection (FBP). We assume that the effect of reconstruction parameters could be significant and potentially lead to the extraction of “fake features.” In this study, we assessed the robustness of radiomic features in DaT-SPECT imaging with different reconstruction parameters using an anthropomorphic head phantom with tissue heterogeneity.

## Materials and methods

### Phantom preparation

An anthropomorphic head phantom with tissue heterogeneity was made using a personal 3D printer. For the phantom preparation, we used CT 1mm-slice images of the head of the primary investigator. The head was scanned by a dual source Somatom Drive CT scanner (Siemens Healthineers, Erlangen, Germany) with the standard acquisition parameters (tube voltage 120 kV, tube current 383 mA, reconstructed with slice thickness 1mm, and pixels 512 × 512). Then, CT images were imported to 3D Slicer (http://www.slicer.org) for the segmentation [23]. The Otsu threshold discriminant analysis method was used to segment the subject’s bones (200 HU ≤ bone region); segmentation was reviewed and edited by an experienced radiologist before the 3D model was created. Following 3D printing was performed on the original Prusa MINI (Prusa Research a.s., Czech Rep.), with polylactide filament (PLA 3D filament, physical density 1.24 g/cm3, Aurapol s.r.o. Czech Rep.) as printing material for soft tissue with 82% infill. PLA was chosen as the soft tissue material primarily due to its reasonable attenuation properties; in comparison to other materials [e.g., Acrylonitrile Butadiene Styrene (ABS), Polyethylene Terephthalate Glycol (PETG)], PLA shows high-level agreement of mass attenuation coefficient with human soft tissue [24]. This infill value of PLA is considered to be reasonable for reproducing human soft tissue according to its HU value in comparison to the Rando phantom (Alderson Radiation Therapy Phantom), which is known to be equivalent to the human body in terms of X-ray absorption and scattering. According to a recent study, the mean HU values of the Rando phantom are -22.5 HU for soft tissue; the soft tissue phantom can be printed with a -20 HU value by using an 82% infill value with a high dice similarity coefficient (DSC = 0.9) [25]. Therefore, in an attempt to replicate the human head attenuation profile more precisely, plaster was used for bone tissue [26]. Plaster powder and water were combined in a 2:1 ratio to make liquid plaster, poured inside the phantom’s hollow to replicate bone. The physical density of the application was approximately 2.3 g/cm^3^. The mixture then hardened for 36–48 hours. A new cavity in the supposed region of basal ganglia was made. A cotton ball with approximately 10 MBq of SPECT radiotracer (^123^I-ioflupane) was inserted; 10 MBq corresponds to the approximal brain 5% uptake after 3–4 hours from the injection to the patient.

### Data acquisition

Scans were performed on the two-detector hybrid camera (GE Optima NM/CT640) with the following acquisition parameters corresponding to international guidelines for DaT SPECT imaging: rotational radius of 11 cm, photopeak 159 KeV ± 10%, matrix 128 × 128, zoom 1.33, angular step 3°, frame time 40 s. Total detected events were > 1.5 million total counts. The phantom’s low-dose CT scan (tube voltage 120 kV, tube current 20 mA, pitch 1, slice thickness 2.5 mm, pixels 512 × 512) was performed to calculate the attenuation correction (AC) map.

### Data reconstruction

SPECT reconstruction was performed on a workstation (GE HealthCare, Xeleris) using ordered subset expectation maximization (OSEM) reconstruction with and without AC and FBP algorithms. Iterative reconstruction was performed using different EM-equivalent iterations shown in Table 1. Reconstruction was carried out using the combinations of iterations and subsets to achieve 20, 40, 60, 80, 100, 150, and 200 EM-equivalent iterations as possible clinical reconstruction strategies. An EM-equivalent iteration is the product of the number of subsets and iterations; for example, two subsets and 20 iterations equals 80 EM-equivalent iterations [27]. OSEM reconstructed data had no post-filtering applied (i.e., post-reconstruction smoothing), which is generally used in clinical settings for better visual assessment. Although radiomic features extracted from CT were robust to low-pass filtering, it could have been a potential source of bias [28]. Butterworth filter with a power factor of 10 and a 0.5 cycles/cm cut-off was used to prefilter FBP data as a local center clinical recommendation for FBP reconstruction of DaT-scan.

**Table 1:** Ordered subset expectation maximization reconstruction strategies.

	Iterations	Subsets	EM—equivalent iterations
**OSEM 1**	2	10	20
**OSEM 2**	4	10	40
**OSEM 3**	6	10	60
**OSEM 4 **	8	10	80
**OSEM 5 **	10	10	100
**OSEM 6 **	10	15	150
**OSEM 7 **	10	20	200

This table displays the iterations and subsets utilized to reach the specified number of EM-equivalent iterations [27]. EM = expectation maximization; OSEM = ordered subset expectation maximization.

### Segmentation and feature extraction

Reconstructed data was uploaded to open-source LifeX software, an International Biomarker Standardization Initiative (IBSI)-compliant and validated software package [29, 30]. In total, 164 features were extracted from the region of interest (ROI) separately for each reconstruction and attenuation correction variant, including size and shape features, intensity features, histogram features (HISTO), Grey-level co-occurrence matrix (GLCM), Grey-level run length matrix (GLRLM), Neighborhood grey-level dependence matrix (NGLDM), and Grey-level zone length matrix (GLZLM). After data cleaning, we ended up with 134 features. A list of extracted features used in this study can be found in supplementary materials (S1 Table).

### Statistics

Statistical analysis was made in the RStudio software [31]. *Psych*.*package* was used for intraclass correlation coefficient (ICC) analysis to assess the repeatability rate for radiomic features in different reconstruction settings based on a two-way mixed effect, fixed raters [32]. ICC was calculated via the following equation:

$$ICC3=\frac{\left(MSR-MSE\right)}{\left(MSR+(k-1)\times MSE\right)}$$

MSE = mean square for error, MSR = mean square for rows, k = number of raters/measurements.

Based on available publications, we decided to interpret ICC in the following manner: an ICC value below 0.50 is considered a sign of poor reliability, 0.50 to 0.75 is moderate reliability, 0.75 to 0.90 good reliability, and a value above 0.90 indicates excellent reliability [33].

The coefficient of variation (COV) was calculated for each feature over the different reconstruction settings via the following equation:

$$COV=\frac{SD}{Mean\times 100}$$

The SD is the standard deviation of feature value, and the Mean is its mean over applying different reconstruction settings.

## Results

**Fig 1:**
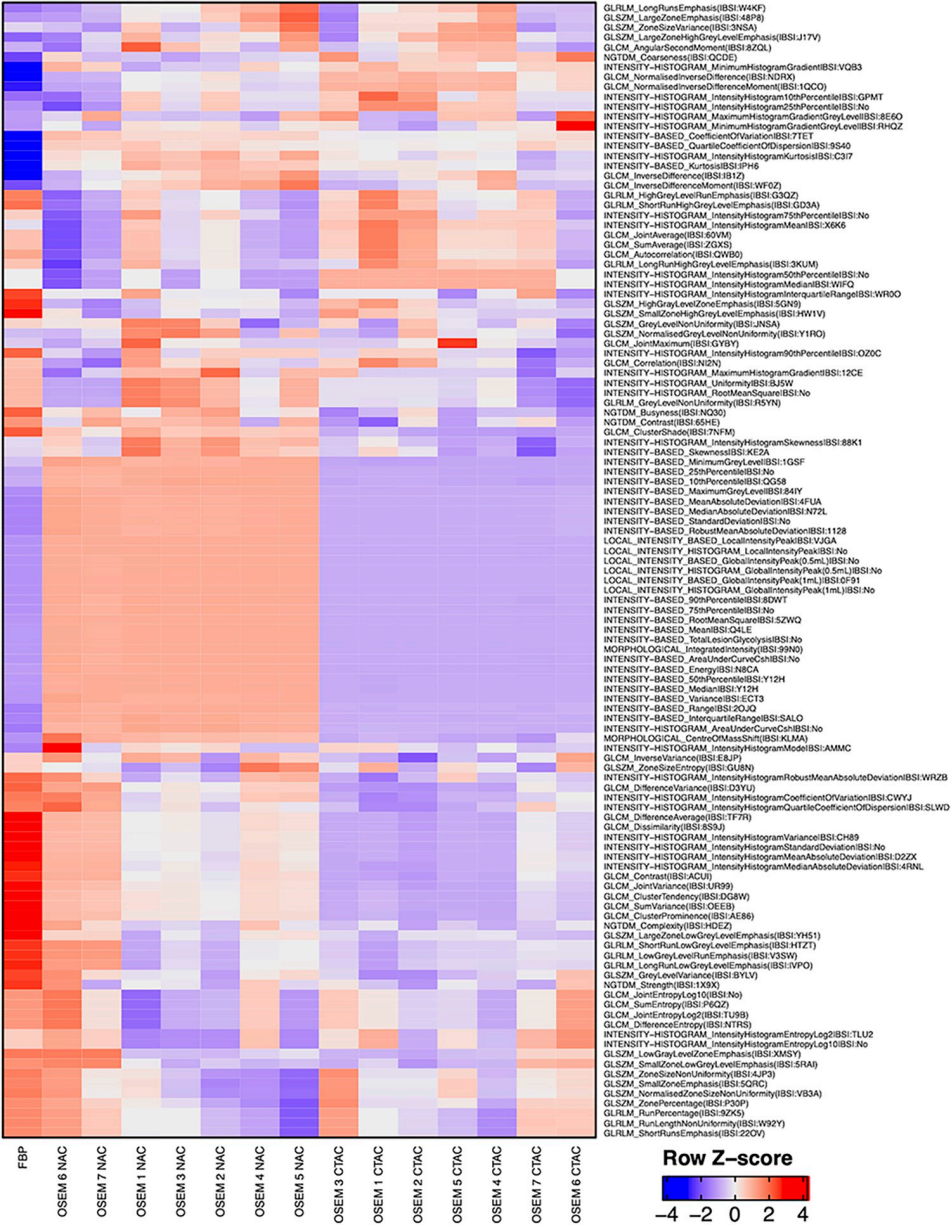
Radiomic features heatmap. This heatmap of the z-score shows the heterogeneity of radiomic features values according to different types of reconstruction. CTAC = CT-based attenuation correction; FBP = filtered back projection; NAC = no attenuation correction; OSEM = ordered subset expectation maximization.

The results describing the impact of reconstruction settings are presented in the radiomic features heatmap (Fig 1), which shows a relatively low variation of z-score value within two main groups, CTAC and NAC. The most pronounced differences in z-score show FBP reconstruction compared to OSEM algorithms.

Overall radiomic features in different reconstruction parameters showed a moderate reproducibility rate with ICC 0.636 [p <0.01, 95% CI (0. 5769177–0. 6960375)]; however, assessment of ICC within CTAC and NAC groups showed excellent reproducibility rate, ICC 0.999 [p <0.01, 95% CI (0.9998476–0.9999095)] and ICC 0.999 [p <0.01, 95% CI (0.9998985–0.9999397)] accordingly.

Reproducibility for each group of radiomic features was different, with the highest reproducibility rate ICC 0.998 [p <0.01, 95% CI (0.9973693–0.9996068)] in GLRLM features, and the lowest reproducibility rate ICC 0.636 [p <0.01, 95% CI (0.4996938–0.7800817)] in intensity-based features (Table 2). However, ICC analysis for each group of radiomic features within the CTAC and NAC reconstruction method showed an excellent reproducibility rate (ICC > 0.9), except for an intensity-based NAC group, where radiomic features of this group showed a good repeatability rate—ICC 0.893 [p <0.01, 95% CI (0.8255056–0.9444637)].

**Table 2:** Reproducibility analysis results.

Radiomic features		ICC3	p value	Confidence interval	
All groups		0.636	< 0.01	0.5769177	0.6960375
	All groups CTAC	0.999	< 0.01	0.9998476	0.9999095
	All groups NAC	0.999	< 0.01	0.9998985	0.9999397
Intensity-based		0.636	< 0.01	0.4996938	0.7800817
	Intensity-based CTAC	0.999	< 0.01	0.9997910	0.9999415
	Intensity-based NAC	0.893	< 0.01	0.8255056	0.9444637
Intensity-histogram		0.844	< 0.01	0.7744021	0.9040791
	Intensity-histogram CTAC	0.999	< 0.01	0.9999225	0.9999726
	Intensity-histogram NAC	0.939	< 0.01	0.9057114	0.9644596
Grey-Level Zone Length Matrix (GLZLM)		0.985	< 0.01	0.9723765	0.9938902
	Grey-Level Zone Length Matrix (GLZLM) CTAC	0.988	< 0.01	0.9772607	0.9953086
	Grey-Level Zone Length Matrix (GLZLM) NAC	0.981	< 0.01	0.9634075	0.9922507
Grey-Level Run Length Matrix (GLRLM)		0.998	< 0.01	0.9973693	0.9996068
	Grey-Level Run Length Matrix (GLRLM) CTAC	0.999	< 0.01	0.9981568	0.9997458
	Grey-Level Run Length Matrix (GLRLM) NAC	0.998	< 0.01	0.9967815	0.9995468
Neighborhood Grey-Level Difference Matrix (NGLDM)		0.984	< 0.01	0.9516552	0.9980031
	Neighborhood Grey-Level Difference Matrix (NGLDM) CTAC	0.998	< 0.01	0.9929343	0.9997529
	Neighborhood Grey-Level Difference Matrix (NGLDM) NAC	0.979	< 0.01	0.9345679	0.9975136
Grey Level Co-occurrence Matrix (GLCM)		0.987	< 0.01	0.9774207	0.9932426
	Grey Level Co-occurrence Matrix (GLCM) CTAC	0.999	< 0.01	0.9984954	0.9995786
	Grey Level Co-occurrence Matrix (GLCM) NAC	0.990	< 0.01	0.9829607	0.9951181
Morphological		0.849	< 0.01	0.7419802	0.9346857
	Morphological CTAC	0.999	< 0.01	0.9999531	0.9999910
	Morphological NAC	0.946	< 0.01	0.8966180	0.9783044

This table shows the results of intraclass correlation coefficient analysis for the different radiomic feature classes. CTAC = CT attenuation correction; ICC = intraclass correlation coefficient; NAC = no attenuation correction.

**Fig 2:**
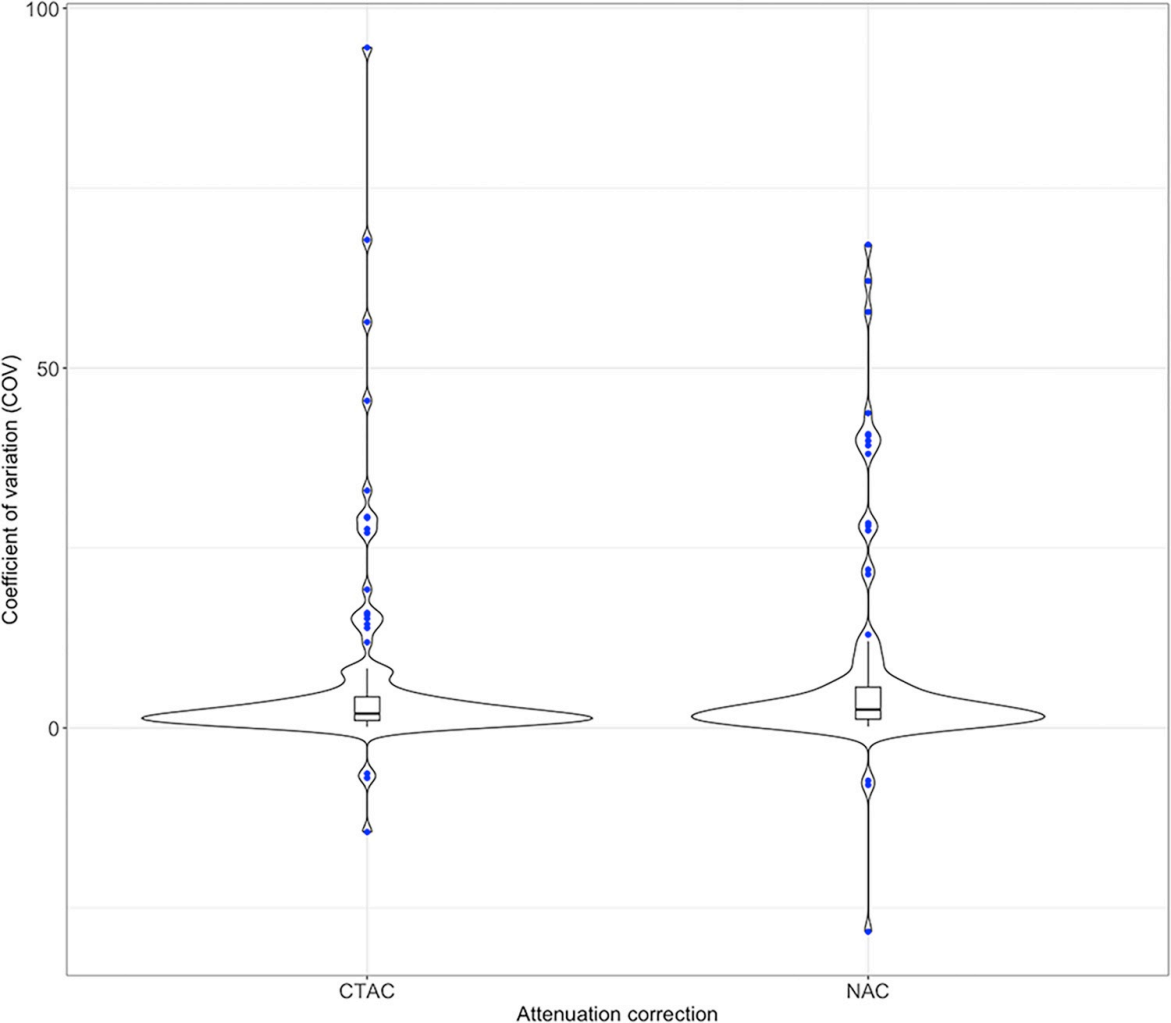
COV density plot. This plot is showing density distribution of coefficient of variation values within two groups—CT attenuation correction and no attenuation correction group; each group contains COV of radiomic features extracted from different reconstruction settings (OSEM1 –OSEM7), radiomics features that showed a significantly higher rate of COV (outliers) are demonstrated by blue dots. Listed outliers can be found in Table 3. CTAC = CT attenuation correction; NAC = no attenuation correction.

Subsequent analysis of the stability of each radiomic feature was made by COV, where most features showed relatively low COV, particularly within CTAC and NAC groups (Fig 2). Detailed results of COV for each radiomic feature can be found in the S1 Table. We identified 22 radiomic features significantly sensitive to the number of EM-equivalent iterations irrespective of the attenuation correction (Table 3).

**Table 3:** List of the least reproducible radiomic features.

Radiomic features	COV in CTAC	COV in NAC
MORPHOLOGICAL Centre Of Mass Shift (IBSI:KLMA)	13.90927	-
INTENSITY-BASED Kurtosis(IBSI:IPH6)	-6.323279	-7.303177
INTENSITY-HISTOGRAM Intensity Histogram Kurtosis (IBSI:C3I7)	-6.920497	-7.905919
INTENSITY-HISTOGRAM Intensity Histogram 10^th^ Percentile (IBSI:GPMT)	19.24501	22.009749
INTENSITY-HISTOGRAM Intensity Histogram Mode (IBSI:AMMC)	29.34633	67.148021
INTENSITY-HISTOGRAM Maximum Histogram Gradient (IBSI:12CE)	14.43376	21.347814
INTENSITY-HISTOGRAM Minimum Histogram Gradient (IBSI:VQB3)	-14.43376	-28.284271
INTENSITY-HISTOGRAM Maximum Histogram Gradient Grey Level (IBSI:8E6O)	67.81306	62.102732
INTENSITY-HISTOGRAM Minimum Histogram Gradient Grey Level (IBSI:RHQZ)	94.52572	39.264521
GLCM Joint Maximum (IBSI:GYBY)	32.99144	28.463752
GLCM Inverse Variance (IBSI:E8JP)	45.46638	27.450501
GLRLM Low Grey Level Run Emphasis (IBSI:V3SW)	15.68774	40.615955
GLRLM Short Run Low Grey Level Emphasis (IBSI:HTZT)	15.99819	40.838739
GLRLM Long Run Low Grey Level Emphasis (IBSI:IVPO)	15.20387	39.893329
GLSZM Low Gray Level Zone Emphasis (IBSI:XMSY)	29.19911	43.747341
GLSZM Small Zone Low Grey Level Emphasis (IBSI:5RAI)	56.39876	57.802980
GLSZM Large Zone Low Grey Level Emphasis (IBSI:YH51)	27.12484	38.096129
GLSZM Large Zone High Grey Level Emphasis (IBSI:J17V)	11.91373	11.671122
GLSZM Zone Size Variance (IBSI:3NSA)	27.64259	28.078152
INTENSITY-HISTOGRAM Intensity Histogram 25thPercentile (IBSI:No)	-	12.06986
GLSZM Zone Size Non Uniformity (IBSI:4JP3)	-	12.97808

This table lists radiomics features that showed the significantly higher rate of coefficient of variation (expressed in %) with and without attenuation correction. CTAC = CT attenuation correction; COV = coefficient of variation; NAC = no attenuation correction.

## Discussion

In this study, we investigated the reproducibility of radiomic features of the DaT SPECT scan concerning different types of image reconstruction using an anthropometric phantom. Our data indicated that overall radiomic features present moderate variations in SPECT images reconstructed with different parameters. The most pronounced difference in z-score shows FBP reconstruction compared to OSEM algorithms. These results were expected, as reconstructing SPECT data using OSEM offers better hot-spot imaging than FBP with none of the characteristic image “banding” seen with the FBP approach [34].

Overall, radiomic features in different reconstruction parameters showed a moderate reproducibility rate; however, the assessment of ICC excluding FBP within CTAC/NAC groups and particular feature classes showed an excellent reproducibility rate. By our results, CTAC becomes the main threat to feature stability. CTAC in DaT-SPECT is not commonly used in clinical practice but could be implemented in research protocols or other SPECT imaging. Therefore, in radiomics studies, images reconstructed with CTAC should not be mixed with NAC images in one dataset. Subsequently, radiomic feature stability was assessed by COV, where most of the features showed relatively low COV, particularly within CTAC and NAC groups (Fig 2). Descriptors of the relationships between image voxels (GLCM, GLRLM, GLZLM, NGTDM) derived textures showed the highest rate of repeatability (ICC > 0.95) and low COV with few exceptions. Those findings are promising for future studies in patients with PS and pose the potential for a clinical translation. Nonetheless, our study confirmed that many radiomic features (22 of 134) were sensitive to the selected reconstruction algorithm by showing a significant COV. Therefore, using these features in diagnostic or predicting radiomics models could produce less reliable results.

However, it is known that different image quality is a main source of irreproducibility and concern in radiomics analysis [35]. In general, there are two sources of variation in the quality of medical images, machine-dependent (e.g., acquisition and image reconstruction/post-processing) and patient-dependent factors (e.g., movement artifacts). Acquisition parameters directly impact the signal-to-noise ratio and, thus, the “texture” of the image [36]. Different reconstruction settings (FBP versus OSEM, number of iterations, and subsets in OSEM) affect the final image as well [37]. One major issue with iterative reconstruction techniques is that after reconstruction, voxel values may not approach convergence and hence show a local bias [34]. Current standard clinical recommendations for dopaminergic imaging state that typically about 100 EM-equivalent iterations should be used [7], suggesting that the discrimination power of the measure is more significant with this value [27], even though increasing the number of iterations leads to increased noise [38]. There were no specific recommendations on equivalent iterations before those publications, and such high EM-equivalent iterations are not common in regular clinical reconstruction settings. Nonetheless, if normal databases are used for semi-quantification, reconstruction parameters should be used according to the database, as semi-quantitative values are affected. On the other hand, results of a recent study on PET image datasets suggest that noise has a significantly high impact on the robustness of the texture analysis [39]. Moreover, image quality is significantly impacted by ROI segmentation, a crucial step in radiomics analysis [40]. The uncertainties in ROI delineation impact the derived radiomic features because all calculations are made on the voxels within the ROI. The “shape” features, in particular, are a direct depiction of the ROI’s qualities and are mostly determined by the ROI’s size and form. Many radiomic features have been demonstrated to be sensitive to ROI delineation in PET imaging [16–18, 41–43].

Blinder et al. investigated advanced texture analysis in quantitative brain imaging. They found strong evidence that such measures retain their information even as one transitions from the higher resolution domain of PET images to the lower resolution domain of SPECT images by a significant post-reconstruction blurring of PET images (e.g., up to 1 cm) [44]. In the following research on DaT SPECT radiomics, they discovered a significant correlation between radiomic features and clinical assessment results, e.g., clinical, motor, and cognitive outcomes [13]. So far, to our knowledge, there have been no studies of the robustness of radiomic features in DaT SPECT.

There is growing interest in measuring uptake heterogeneity by textural analysis in DaT SPECT and other imaging modalities as potential diagnostic, predictive, and prognostic biomarkers. However, we should understand the precision of these measurements and the effects of different processing and analytic methods before they become more widely used, particularly in the multi-center study setting. Radiomic features with high ICC and low COV can be considered good candidates for reproducible analysis (e.g., multi-center studies), and standardization is the key to successful clinical implementation of texture analysis.

Our study has several limitations. We used image datasets acquired with a head phantom, and despite our best efforts to make an anthropomorphic head phantom with heterogeneous tissue imitating human head attenuation, we could not ensure radiopharmaceutical distribution similar to patient images. This needs to be overtaken by future research with real patient image data. In this study, we omitted post-reconstruction smooth filtering as a source of potential bias, although radiomic features extracted from CT were robust to low-pass filtering [28]. Based on experience with quantitative SPECT, we assume post-filtering is a significant source of negative bias, as the post-reconstruction smoothing filter decreases the spatial resolution and amplifies partial volume effects [27]; in other words, “smoothing is wiping out the texture.” However, precise data on SPECT image datasets are lacking. Another limitation of this study was that the implementation of the OSEM algorithm from only one vendor was used. Software audits have shown that some image variations may occur with different software algorithms [45], thus having a prominent impact on radiomic features stability because of inter-vendor image variability. Accurate delineation of the volume of interest (VOI) is crucial for the computation of radiomic features; therefore, one of the other limitations was the usage of fixed VOI. In an attempt to exclude the potential bias of inconsistent delineation of ROI, fixed VOI significantly influenced the stability of “shape” features. However, a recent study investigating metabolic VOI segmentation showed that the variation in image segmentation thresholds only has minor effects on the quantification [21].

## Conclusions

According to our results, radiomic features extracted from DaT SPECT showed moderate to excellent repeatability rates within different reconstruction settings. The primary source of variation in values of radiomic features is reconstruction algorithms with and without attenuation correction (CTAC and NAC). However, many radiomic features were sensitive to the selected reconstruction algorithm irrespectively to the attenuation correction. These results make radiomic features suitable for clinical practice and human studies, but awareness of feature selection should be held, as some radiomic features are more robust than others.

## Supporting information

S1 Table COV of radiomic features in different reconstruction settings. This table lists the coefficient of variation (expressed in %) for each extracted radiomic feature between different reconstruction settings (OSEM1—OSEM7) within two groups, with (CTAC) and without (NAC) attenuation correction.
(XLSX)
